# Institutionelle Entwicklungslinien der Anti-Doping-Politik des Internationalen Olympischen Komitees: Historische und gegenwärtige Perspektiven

**DOI:** 10.1007/s00103-026-04248-0

**Published:** 2026-05-21

**Authors:** Stephan Wassong

**Affiliations:** Institut für Sportgeschichte, Zentrum für Olympische Studien, Deutsche Sporthochschule Köln, 50968 Köln, Nordrhein-Westfalen Deutschland

**Keywords:** IOC Sub-Komitee Doping, Medizinische Kommission IOC, Welt-Anti-Doping-Agentur, Nationale Anti-Doping-Agenturen, International Testing Agency, Doping-Skandale, IOC Doping Subcommittee, IOC Medical Commission, World Anti-Doping Agency, National Anti-Doping Agencies, International Testing Agency, Doping scandals

## Abstract

Die Nutzung von leistungsfördernden Substanzen lässt sich bis vor den Beginn der ersten Austragung der Olympischen Spiele der Neuzeit zurückverfolgen. Das 1894 gegründete Internationale Olympische Komitee (IOC) erstellte 1938 erstmals Handlungsempfehlungen zum Doping von Athlet:innen. Darin wurde Doping verurteilt und ein Ausschluss betroffener Athlet:innen vorgesehen. Seit den 1960er-Jahren wurden zunehmend Institutionen zur Bekämpfung des Dopings im Sport aufgebaut. Die Regelungen wurden präziser und 1968 wurden bei Olympischen Winterspielen in Grenoble erstmals Doping-Kontrollen durchgeführt.

Der Artikel bietet einen Überblick über die Entwicklung des institutionellen Anti-Doping-Kampfs, mit besonderem Fokus auf das Doping-Sub-Komitee des IOC, die Medizinische Kommission (MK) des IOC, die Welt-Anti-Doping-Agentur (WADA), die Nationalen Anti-Doping-Agenturen (NADAs) und die International Testing Agency (ITA). Darüber hinaus werden die aktuellen Herausforderungen des Anti-Doping-Netzwerks im Zusammenhang mit den privat geplanten „Enhanced Games“ beleuchtet, bei denen Doping ausdrücklich erlaubt ist.

## Abstract (en)

The use of performance-enhancing substances can be traced back to before the first modern Olympic Games were held. The International Olympic Committee (IOC), founded in 1894, issued its first guidelines on doping in 1938. These guidelines condemned doping and proposed the exclusion of athletes who consumed performance-enhancing substances. Since the 1960s, institutions dedicated to combating doping in sport have been continuously established. Regulations became more precise, and in 1968, doping controls were conducted for the first time at the Winter Olympics in Grenoble.

This article provides an overview of the development of the institutional anti-doping network, with a particular focus on the IOC’s Doping Subcommittee, the IOC Medical Commission (MC), the World Anti-Doping Agency (WADA), the National Anti-Doping Agencies (NADAs), and the International Testing Agency (ITA). In addition, the article examines the current challenges facing the anti-doping network in connection with the privately organized “Enhanced Games,” in which doping is explicitly permitted.

## Einleitung

Im Jahr 1894 wurde das Internationale Olympische Komitee (IOC) gegründet, um die ersten Olympischen Spiele der Neuzeit zu initiieren und deren erste Austragung 1896 in Athen zu organisieren. In den ersten Jahrzehnten nahmen vornehmlich Amateursportler:innen an den Olympischen Spielen teil. Ab den 1980er-Jahren – und damit bereits zu Beginn der IOC-Präsidentschaft von Juan Antonio Samaranch – wurden die Olympischen Spiele zunehmend auch für Profisportler:innen geöffnet und die Vermarktung des olympischen Sports systematisch ausgebaut [1]. Diese beiden Strategieentscheidungen des IOC markieren jedoch noch nicht den Beginn der Doping-Problematik im olympischen Sport. Die Nutzung von leistungsfördernden Substanzen lässt sich bis vor die erste Austragung der Olympischen Spiele der Neuzeit zurückverfolgen. Damals wurden Athlet:innen noch nicht getestet; weshalb auch keine Sanktionen erfolgen konnten [2].

Ziel des Artikels ist es, aufzuzeigen, wann und wie sich das IOC der Verpflichtung gestellt hat, erste Anti-Doping-Initiativen auf institutioneller Ebene aufzubauen. Im Anschluss daran folgt eine Analyse zu der Frage, warum und in welcher Art und Weise das IOC das in den 1960er-Jahren gegründete institutionelle Anti-Doping-Netzwerk kontinuierlich erweitert hat. Die Gliederung orientiert sich an den Zeiträumen 1930 bis 1959, 1960 bis 1998, 1999 bis 2014 und 2015 bis zur Gegenwart. Abschließend werden aktuelle Herausforderungen des Anti-Doping-Netzwerks beleuchtet.

## 1930 bis 1959: Anfänge der institutionellen Doping-Bekämpfung

Bereits ab Mitte der 1930er-Jahre hatte sich das IOC den Bemühungen der *International Amateur Athletic Association *(IAAF und heute *World Athletics) *angeschlossen, den zunehmend sichtbaren Konsum von leistungssteigernden Substanzen zu untersuchen und zu sanktionieren [3]. Es war kein Zufall, dass der Engländer Lord Burghley die Anti-Doping-Diskussionen im IOC anstieß, da er nicht nur IOC-Mitglied war, sondern auch verschiedene Funktionärsämter in der *Amateur Athletic Foundation*, dem Dachverband der englischen Leichtathletik und institutionelles Mitglied der IAAF, bekleidete. Darüber hinaus war Burghley ein erfolgreicher Leichtathlet, der an den Olympischen Spielen 1924 in Paris, 1928 in Amsterdam und 1932 in Los Angeles teilnahm [4]. 1924 gewann er sogar Gold über die 400-Meter-Strecke.

Auf Vorschlag von Burghley wurde auf der IOC-Session in Warschau 1937 eine erste Arbeitskommission zur Doping-Problematik gegründet. Im nächsten Jahr erfolgte auf der IOC-Session in Kairo die Vorlage von Arbeitsergebnissen, deren Ziel es war, Regeln gegen Doping-Missbrauch vorzuschlagen. Die im Folgenden zitierten Handlungsempfehlungen, die mit denen der IAAF aus dem Jahr 1928 nahezu identisch waren, wurden zwar im Protokoll der Session, aber nicht in der Olympischen Charta niedergeschrieben:

„The use of drugs or artifical stimulants of any kind must be condemmed most strongly, and anyone who accepts or offers dope, no matter in what form, should not be allowed to participate in amateur meetings or in the Olympic Games“ [5].

Wie der Auszug aus der Handlungsempfehlung zeigt, wollte man dopende Sportler – die man vor allem im Profisport verortete – strikt vom olympischen (Amateur‑)Sport fernhalten. Das Sporttreiben der Profis wurde von Mitgliedern im IOC pauschal kritisiert, allen voran vom Begründer der modernen Olympischen Spiele, dem französischen Gelehrten Pierre de Coubertin. Dem Profisport wurde keine moralische Wertigkeit zugesprochen, „da dessen Zielsetzung im Gelderwerb angelegt sei. Durch diese extrinsische Motivation werden für Coubertin [und andere Sportoffizielle] Fehlentwicklungen im Sport manifestiert, die sich unter anderem in einem übertriebenen Konkurrenzverhalten, Unfairness und sogar Manipulationen“ [6] von Leistungen zeigen können. Vor diesem Hintergrund zielte die vom IOC vorläufig verfasste Handlungsempfehlung darauf ab, Doping als Gefahr für die Chancengleichheit im Sport und die Gesundheit der Athleten zu verurteilen. Welche Substanzen oder Methoden allerdings als Doping bezeichnet wurden, wurde nicht aufgelistet.

In den Folgejahrzehnten wurden keine weiteren Schritte zur Umsetzung der genannten Leitlinie von 1938 unternommen. Die Begründungen dafür sind nur teilweise mit dem Ausfall der Olympischen Spiele 1940 und 1944 und auf den mühevollen Wiederaufbau der Olympischen Bewegung nach 1945 zu erklären. Fakt war vielmehr: Das IOC sah Doping noch nicht als ernst zu nehmende Bedrohung für die Aushöhlung der Integrität des olympischen Sports an, da dieser ja rein auf dem Amateurwesen basieren sollte. Eine Änderung dieser Position war erst ab den 1960er-Jahren spürbar.

## 1960 bis 1998: Auf- und Ausbau der Medizinischen Kommission des IOC

Auslöser dafür war der Tod des dänischen Radfahrers Knud Enemark Jensen bei den Olympischen Spielen 1960 in Rom. Jensen war beim 100-Kilometer-Mannschaftszeitfahren gestürzt und anschließend im Krankenhaus verstorben. Die Autopsie hatte ergeben, dass Jensen Amphetamine eingenommen hatte, wodurch er sich im Wettbewerb wahrscheinlich nicht so erschöpft gefühlt hatte, wie er wirklich war. Der dadurch herbeigeführte Kollaps war die Ursache für den Sturz, der dann zu tödlichen Gehirnverletzungen führte. Als Konsequenz dieses tragischen Ereignisses richtete das IOC ein Doping-Sub-Komitee im Jahr 1962 ein. Es stand unter der Leitung von Arthur Porritt, einem neuseeländischen IOC-Mitglied. Er war zudem Präsident des *Royal College of Surgeons of England *[3]*.*

Die Aktivitäten von Porritt zum Aufbau von Anti-Doping-Leitlinien waren allerdings nicht sehr umfassend. Das hing auch mit der Zurückhaltung von IOC-Präsident Avery Brundage in Bezug auf den Anti-Doping-Kampf zusammen. Nach Auffassung von Brundage schien die aufkommende Doping-Problematik mit einer konsequenten Haltung des IOC zum Amateurprinzip geregelt werden zu können. Für ihn war auch folgende bemerkenswerte Aussage von Porritt unwichtig. Porritt hob bereits 1966 hervor, „that only a long-term education policy stressing the physical and moral aspects of the drug problem would stop athletes from using performance-enhancing substances“ [7]. Vielleicht war diese Hinwendung zum Präventivpotenzial der Erziehung auch nur durch die Tatsache bedingt, dass es noch keine Nachweisverfahren zur Aufdeckung der Einnahme von leistungsfördernden Substanzen gab.

Porritt schied 1966 aus dem Sub-Komitee aus; er übernahm stattdessen das Amt des Gouverneurs von Neuseeland und war damit dann anderen Aufgaben verpflichtet. Seinem Nachfolger, IOC-Mitglied Alexandre de Merode aus Belgien, hatte Porritt eine vorläufige Liste mit verbotenen Substanzen übermittelt, sodass in der Folgezeit einige Orientierungspunkte im Anti-Doping-Kampf aufgestellt werden konnten.

Merode implementierte eine konsequentere Struktur, um das Vorgehen gegen Doping proaktiver zu gestalten und institutionell effektiver zu positionieren. So wurde 1967 das Sub-Komitee aufgelöst und in die Medizinische Kommission (MK) des IOC überführt [3]. Die Notwendigkeit für diese institutionelle Neuausrichtung, die nun einen offiziellen Kommissionsstatus besaß, wurde durch die tragische Nachricht verstärkt, dass es bei der Tour de France 1967 – schon wie bei den Olympischen Spielen 1960 in Rom – einen Todesfall gegeben hatte: Tom Simpson aus Großbritannien war während der 13. Etappe verstorben. Als Todesursache wurde Erschöpfung angegeben, die durch die Hitze des Tages hervorgerufen wurde und durch die Einnahme von Amphetaminen noch unkontrollierbarer geworden war [8].

Unter der Leitung von Merode wurde darauf gedrängt, das Verbot des Einsatzes von unerlaubten leistungsfördernden Substanzen möglichst schnell in der Olympischen Charta zu verankern. Auf Vorschlag der MK beschloss das IOC folgende Regelungen, die schließlich 1967 ins Regelwerk des IOC aufgenommen wurden:

„The use of drugs or artificial stimulants of any kind is condemned and any person offering or accepting dope, in any form whatsoever, cannot participate in the Olympic Games. Conviction of a competitor for the use of dope shall result in the disqualification of the entire team in that sport“ [9].

Die MK führte bereits bei den Olympischen Winterspielen in Grenoble 1968 Doping-Kontrollen für ausgewählte Substanzen durch. Athlet:innen wurden zunächst auf Alkohol, Amphetamine, Ephedrin und Cannabis getestet. Die Kontrollen verliefen sehr unkoordiniert und es konnten offiziell keine positiven Doping-Befunde erhoben werden. Bei den Olympischen Spielen 1968 in Mexico City verliefen die Doping-Tests ebenfalls unbefriedigend. Das Spektrum an zu analysierenden Substanzen war genauso begrenzt wie in Grenoble [10].

In den Folgejahren setzte die MK ihre Aktivitäten weiter fort, was unter anderem zur Akkreditierung von Anti-Doping-Laboren und zur Entwicklung von neuen Test- und Analyseverfahren führte. Diese sollten sich vor allem auf den Nachweis von anabolen Steroiden konzentrieren, da die Einnahme von Anabolika unter Athlet:innen eine zunehmend gängige Strategie zur Leistungssteigerung war. Gemäß einer anonymen Umfrage, die von dem US-amerikanischen Leichtathleten Jay Silvester (Diskuswerfer und Silbermedaillengewinner bei den Olympischen Spielen 1972 in München) unter allen Leichtathlet:innen durchgeführt worden war, hatten in München 68 % aller Teilnehmer:innen anabole Steroide vor dem Beginn der olympischen Wettkämpfe konsumiert [11]. Bei den Münchner Spielen selbst wurden noch keine offiziellen Anabolika-Tests durchgeführt. Sie wurden seitens der MK erst bei den Olympischen Spielen 1976 in Montreal eingeführt und dann auch auf die Liste der verbotenen Substanzen aufgenommen, die von der MK 1971 offiziell entwickelt worden war [12].

Mit der Einführung von Doping-Kontrollen und der Erstellung der Liste von verbotenen Substanzen zur Leistungssteigerung hat letztlich eine Transformation in der Auslegung der Doping-Definition stattgefunden. Der Sportsoziologe Marcel Reinold fasst dies in seiner Publikation *Doping als Konstruktion. Eine Kulturgeschichte der Anti-Doping-Politik *folgendermaßen zusammen:

„Bis zur Einführung von Kontrollen wurde die Definition nicht für sportrechtliche Belange der Dopingverfolgung konzipiert, sondern sollte die Benutzung pharmakologischer Präparate im Sport verurteilen. Ohne Kontrolle … galt vor allem die Vermittlung der Verwerflichkeit von Doping als wirksame Präventivstrategie. Dafür taugten keine moralisch-neutralen Dopinglisten, denn letztlich kam es weniger auf die benutzte Substanz an als vielmehr – den erzieherischen und moralischen Ideen des Sports entsprechend – auf die richtige Gesinnung der Athleten. … [Durch] den Einzug juristisch-technokratischer Prinzipien wie Dopinglisten [wurde Doping] zu einem technisch vermittelten Konstrukt, bei dem nicht mehr nach der Entscheidung einzelner Personen gefragt wurde, sondern nach den juristischen Tatbeständen, d. h. konkret den biochemischen Parametern“ [13].

Das IOC hatte mit der MK eine hausinterne Institution geschaffen, die nun gegen den Missbrauch von Doping bei Olympischen Spielen ankämpfte. Neben den bereits aufgeführten administrativen und wissenschaftlichen Zuständigkeitsbereichen, in deren Zentrum die Kontrollen bei den Olympischen Spielen und ab 1988 auch die Durchführung von sogenannten Out-of-Competitions-Tests standen, bereitete die MK für das IOC auch Sanktionierungsentscheidungen für Verstöße gegen die Doping-Regeln vor [14].

Aber trotz der Positionierung der MK im Anti-Doping-Kampf müssen die Doping-Statistiken über positiv getestete Athlet:innen doch mit großer Vorsicht betrachtet werden. Im Zeitraum zwischen 1968 und 1998 wurden bei den Olympischen Spielen nur 53 Athlet:innen positiv getestet (48 bei den Olympischen Sommer- und 5 bei den Olympischen Winterspielen; [15]). Diese geringe Anzahl verwundert nur auf den ersten Blick. Denn in jenen 30 Jahren lag die Hochphase des Kalten Krieges. Der Sport und vor allem die Olympischen Spiele waren damals ein Mittel zum Zweck, nationale Stärke und Block-Größen abzubilden.

Methoden zur Verschleierung des Konsums von Substanzen waren auch damals schon bekannt. Die Einführung von Trainingskontrollen erwies sich als wenig effektiv, unter anderem, weil der Zugang von Kontrolleur:innen Athlet:innen zu Wettkampfstätten unter den sportpolitischen Rahmenbedingungen des Kalten Krieges oftmals nicht gewährt wurde. Darüber hinaus wurde – teils unter politischer und sportverbandspolitischer Patronage – von Sportmediziner:innen die Leistungssteigerung oftmals höher gewichtet als die ethische Verantwortung für die Gesundheit der Athlet:innen. Das beruflich erworbene Wissen über pharmakologische Entwicklungen leistungssteigernder Mittel vergrößerte die Diskrepanz zwischen der Anwendung von Doping-Mitteln und den entsprechenden Nachweisverfahren [16].^1^

Das IOC zeigte sich bis zum Ende der 1990er-Jahre zufrieden mit der Handhabung der Statistiken. Die Tatsache, dass bei den Olympischen Spielen selbst nur wenige positive Fälle auftraten, einschließlich positiver Tests von Spitzenathleten wie Ben Johnson (100-Meter-Finale 1988 in Seoul), schien als Nachweis für die Wirksamkeit der internen Anti-Doping-Strategien ausreichend und überzeugend zu sein [17]. Das IOC hatte wenig Interesse daran, die Doping-Problematik über die Olympischen Spiele hinaus zu betrachten. Es übernahm für die Wettkämpfe der internationalen Sportverbände und die dort notwendigen Doping-Tests keine Verantwortung [10]. Im Jahr 1998 jedoch änderte sich diese Einstellung nachhaltig.

## 1999 bis 2014: Neuausrichtung etablierter institutioneller Strukturen im Anti-Doping-Kampf

Bei der Tour de France 1998 leitete die französische Staatsanwaltschaft große Razzien in den Mannschaftshotels und Teamautos der Festina-Mannschaft ein. Schon vor dem Start der Tour wurden im Pkw eines Physiotherapeuten große Mengen an verbotenen Doping-Substanzen wie Steroide, Amphetamine und Erythropoetin (EPO) gefunden, das zu jener Zeit das wichtigste Doping-Mittel war. Eine Detektierung der Substanz war 1998 noch nicht möglich und ein valides Testverfahren für die Zuführung körperfremder und synthetisch hergestellter EPOs etablierte sich erst ab 2001. Das beim Festina-Team systematisch betriebene Doping führte zum umgehenden Ausschluss des Radteams aus der laufenden Tour und deckte zugleich Doping-Missbrauch bei anderen Radsportteams auf [8].

Obwohl der Doping-Skandal nicht bei den Olympischen Spielen auftrat, richtete sich das mediale und öffentliche Unverständnis auch gegen das IOC. Als Ansatzpunkt galt, dass die Aufdeckung des Festina-Skandals durch staatliches Eingreifen ausgelöst worden ist und nicht durch Handlungsinitiativen, die vom IOC gegen die Anwendung von unerlaubten leistungsfördernden Substanzen und Methoden ergriffen worden waren. Erneut wurde offensichtlich, dass in der Kultur des Spitzensports – und damit auch in dem der Olympischen Spiele – die Bedeutung des Leistungserfolges den Wert der Gesundheit überlagerte. Für die Öffentlichkeit ging dies mit einem Imageverlust des olympischen Sports einher. Kritisiert wurde das Selbstverständnis des IOC, als Weltregierung des Sports aufzutreten, die sich gleichzeitig aber als naiv und machtlos im Kampf gegen Doping zeigte.

Als Reaktion auf die ansteigende kritische Öffentlichkeit und zur Wiedererlangung seiner moralischen Autorität organisierte das IOC vom 02. bis zum 04.02.1999 die *1*^*st*^*World Conference on Doping in Sport *in Lausanne, um zusammen mit Sportfunktionär:innen, Athlet:innen, Politiker:innen und Wissenschaftler:innen Maßnahmen für ein internationales Vorgehen gegen Doping zu besprechen. Vor allem Regierungsvertreter wie der damalige deutsche Innenminister Otto Schily, der zu dieser Zeit auch die EU-Ratspräsidentschaft ausübte, und der britische Sportminister Anthony Louis Banks forderten den Aufbau einer vom IOC unabhängigen, autonomen und transparent agierenden Anti-Doping-Behörde [16]. Eine Beteiligung aus der Politik durfte nicht mehr ausgeschlossen werden. Es war notwendig, den im (olympischen) Sport vorliegenden Versäumnissen im Anti-Doping-Kampf die grundrechtliche Verpflichtung der Staaten zum Schutz der Gesundheit von Athlet:innen entgegenzubringen.

Im Ergebnis wurde am 10.11.1999 die Welt-Ant-Doping-Agentur (WADA) gegründet. Als Hauptsitz der Agentur wurde sich symbolisch für Montreal und nicht für Lausanne entschieden, dem Sitz des IOC und vieler internationaler Sportverbände. Es wurde sich darauf verständigt, die Führungs- und Leitungsgremien der WADA paritätisch mit Vertreter:innen aus dem internationalen Sport und Regierungen mit kontinentaler Repräsentanz zu besetzen [18]. Bis heute umfasst die Rolle der Regierungen bei der WADA nicht nur eine Verpflichtungserklärung, sich aktiver und direkter bei der Bekämpfung des Dopings zu engagieren, sondern auch die Bereitstellung entsprechender finanzieller Zuschüsse für die Durchführung der Anti-Doping-Strategien. Das Budget der WADA wird zu jeweils 50 % vom IOC und den kontinentalen Regierungsvertretungen bereitgestellt [19].^2^

Zu den Hauptaufgaben der WADA gehört die Schaffung von Rahmenbedingungen zur Harmonisierung des internationalen Anti-Doping-Kampfes. Es geht dabei vornehmlich um administrative, ethische, infrastrukturelle, rechtliche, politische, gesundheitliche und wissenschaftliche Aufgabenfelder. Im Kern werden diese im *World Anti Doping Code *(WADC) dargestellt. Diesen Code unterschreiben unter anderem internationale Sportfachverbände, nationale olympische und paralympische Komitees, nationale Sportfachverbände und Organisatoren von Großsportveranstaltungen. Es besteht keine generelle Unterschriftsverpflichtung, jedoch werden z. B. internationale Sportfachverbände von Olympischen Spielen ausgeschlossen, wenn keine Unterschrift vorliegt [20].

Als Konsequenz der WADA-Gründung als unabhängige Agentur musste das IOC zentrale Aufgabenbereiche an die WADA übertragen, die zuvor im Verantwortungsbereich der MK lagen. Dies betrifft die Erstellung und Publikation der Liste verbotener Substanzen, die Akkreditierung der internationalen Anti-Doping-Labore sowie die Einführung und Verwaltung individueller medizinischer Datensätze von Athlet:innen. Die Doping-Kontrollen bei den Olympischen Spielen wurden zunächst weiterhin vom IOC in Kooperation mit den jeweiligen Organisationskomitees der Olympischen Spiele durchgeführt [21].

Nach der Etablierung der WADA wurden nationale Anti-Doping-Agenturen (NADAs) aufgebaut, deren institutioneller Status variieren kann. In Frankreich, Russland und Großbritannien ist die NADA eine Regierungsbehörde. Die NADAs in Deutschland und in der Schweiz sind als Stiftungen aufgebaut, die zu einem Großteil von der Regierung unterstützt werden. Die NADA Deutschland wird zu 60 % von der Bundesregierung unterstützt. Die restlichen 40 % können durch Landes- oder Finanzmittel von Sportdachorganisatoren aufgefüllt werden. In Schweden und Italien sind die NADAs institutioneller Teil von nationalen Sportfachverbänden oder nationalen olympischen Komitees.

Derzeit existieren weltweit 209 NADAs, zu deren Hauptaufgaben die Durchführung von Doping-Tests bei Wettkämpfen, Doping-Kontrollen im Training, die Verantwortlichkeit des Ergebnismanagements, die Einleitung von Disziplinarverfahren sowie die präventiv wirkende Aufklärungsarbeit über Doping gehören. Die WADA überprüft die Aktivitäten und Initiativen der NADAs hinsichtlich ihrer Übereinstimmung mit dem WADC. Bei mangelnder Kompatibilität hat die WADA die Möglichkeit, den NADAs ihre Akkreditierung zu entziehen [22].

Durch die institutionelle Neuausrichtung des Anti-Doping-Kampfes sowie die Verteilung und Übertragung von Verantwortlichkeiten wurden offenbar effektive Initiativen ergriffen, um die Doping-Problematik zumindest einzugrenzen. Für das IOC war dies vielversprechend, da „in der Öffentlichkeit seine Verantwortung ebenso wie seine Kompetenzen als Weltregierung des Sports nicht mehr grundsätzlich in Frage gestellt wurden“ [21]. Für diese Annahme konnten die Doping-Statistiken von den Olympischen Spielen als Argumentationsbasis angenommen werden (Tab. 1).

**Tab. 1:** Doping-Statistik bei den Olympischen Spielen von 1996 bis 2012 (Referenzjahr 2013; [21, 27]).

Olympische Spiele	Urinproben	Blutproben	Positive Tests
Atlanta 1996	1947	0	2
*November 1999: Gründung der WADA*			
Sydney 2000	2769	625	11
*Januar 2004: Implementierung des WADC*			
Athen 2004	2926	691	23
Peking 2008	3801	969	14 (inklusive Nachtests)
London 2012	4005	1057	11 (inklusive Nachtests)

*WADA* Welt-Anti-Doping-Agentur, *WADC* World Anti Doping Code

Der Anstieg der positiven Proben nach der Gründung der WADA, den Neueinrichtungen von NADAs und der Einführung des WADC schien die Effektivität der neuen institutionellen Handlungsleitlinien und Verantwortungsbereiche zu bestätigen. Der Rückgang der Überführungsquoten von positiv getesteten Athlet:innen bei den Olympischen Spielen in Peking und London wurde nach außen hin als Nachweis für eine Eingrenzung des Doping-Problems betrachtet.

Es ist allerdings zu beachten, dass sich die hier dargestellten Daten (Tab. 1) auf das Referenzjahr 2013 beziehen. Ab 2014 stieg die Zahl von Doping-Nachweisen erheblich an, was mit der Aufdeckung des großen Doping-Skandals in Russland zusammenhing. Dieser führte nicht nur zu einer Modifikation der Doping-Statistiken, sondern auch zu einer umfassenden Erweiterung und Neustrukturierung des institutionellen Anti-Doping-Netzwerkes.

## 2015 bis zur Gegenwart (2025): Die Ausweitung des interinstitutionellen Anti-Doping-Netzwerkes

Ab 2015 mehrten sich Nachweise über flächendeckendes Doping in Russland, das hauptsächlich im Vorfeld und während der Olympischen Winterspiele in Sotschi 2014, der Olympischen Spiele in London 2012 und Peking 2008 orchestriert worden war. Zentrale Akteure waren dabei die russische Regierung, die russische Anti-Doping-Agentur (RUSADA) und der staatliche Inlandgeheimdienst. Belastende Informationen zu diesen Vorgängen wurden nicht durch analytische Nachweisverfahren, sondern durch Whistleblower^3^ und Investigativjournalisten (z. B. ARD-Fernsehdokumentation „Geheimsache Doping“) ans Licht gebracht. IOC und WADA nahmen diese Informationen an und leiteten entsprechende Maßnahmen ein.

Zunächst beauftragte das IOC eine Sonderkommission damit, die Doping-Proben von den Olympischen Winterspielen in Sotschi 2014 nachzutesten. Das IOC hatte damit das Recht in Anspruch genommen, eingefrorene Doping-Proben bis zu 8 Jahre nach den Olympischen Spielen mit den neuesten biochemischen Nachweisverfahren zu testen. Die Richtlinie dazu wurde bereits nach den Olympischen Spielen in Athen 2004 erstellt. Nach den Olympischen Spielen in Rio 2016 wurde die Frist für Nachtests auf 10 Jahre verlängert. Bis Januar 2018 wurden insgesamt 311 Urin- und Blutproben nachgetestet und 43 Sportler:innen wurden wegen Dopings angeklagt. 30 von ihnen legten erfolgreich Berufung beim Internationalen Sportgerichtshof (*Court of Arbitration for Sport*, CAS) ein. Lediglich bei 12 Athlet:innen wurde die Entscheidung des CAS aufrechterhalten. Ihre zunächst lebenslange Sperre, an den Olympischen Spielen teilzunehmen, wurde jedoch aufgehoben. Die WADA entzog der RUSADA ihre Akkreditierung für die Durchführung von Doping-Tests [21].

Der Doping-Skandal offenbarte aber auch eine Lücke in dem aufgebauten institutionellen Anti-Doping-Netzwerk zwischen IOC, WADA und NADAs. Das Vertrauen in die Unabhängigkeit von NADAs, die direkt einem Sportdachverband angeschlossen und/oder als staatliche Behörde organisiert sind, war erschöpft. Vor allem der zuletzt aufgeführte institutionelle Status rückte ins Zentrum der Kritik.

Seit 2016 berieten das IOC und die WADA über die Einrichtung einer weiteren internationalen Anti-Doping-Agentur, die auf institutioneller Ebene zwischen IOC, WADA und NADAs agieren sollte. Auf diese Weise sollte im Besonderen der Einfluss aus der Politik und seitens der Sportfachverbände weiter reduziert werden. Ziel war es auch, den Kampf gegen Doping transparenter zu gestalten. Diese neue Institution – die *International Testing Agency *(ITA) – wurde 2018 mit Geschäftssitz in Lausanne gegründet [23].

Die weitestgehende Unabhängigkeit der ITA soll personell durch die Zusammensetzung der operativen Vorstandsebene erreicht werden. Vier Mitglieder mit hohen Fachexpertisen in juristischen, ethischen, naturwissenschaftlichen und ökonomischen Angelegenheiten und ohne aktive Tätigkeiten in Sportorganisationen oder in der Politik werden jeweils von einem/einer Repräsentant:in des IOC und den internationalen Sportfachverbänden unterstützt. Die WADA benennt einen sogenannten *Observer, *der nicht stimmberechtig ist. Des Weiteren gehört dem Vorstand ein Mitglied der WADA-Athletenkommission an [24].

Finanziell sollte die ITA unabhängig sein. Vor allem die Subventionen seitens des IOC sollten aus Compliance-Gründen deutlich reduziert werden. Das IOC hat die Anfangsfinanzierung mit 30 Mio. US-Dollar zugesichert. Mittlerweile übernimmt das IOC nur noch 9 % des ITA-Jahresbudgets, das 2025 die Summe von 60 Mio. US-Dollar umfasst. Die Haupteinnahmequellen der ITA stammen von mehr als 70 internationalen Sportverbänden, von Organisationskomitees von Sportgroßveranstaltungen und teilweise auch von NADAs [25].

Die Dienstleistungen der ITA umfassen die Durchführung von Doping-Kontrollen bei nationalen und internationalen Wettkämpfen, die Organisation des Ergebnismanagements der analysierten Proben, das Monitoring von Kontrollen außerhalb der Wettkämpfe, die fachgerechte Lagerung von Doping-Proben über einen Zeitraum von 10 Jahren, die Regulierung der *Therapeutic Use Exemptions *(bestätigte therapeutische/medizinische Erlaubnis für die Einnahme von Medikamenten, die auf der Dopingliste stehen) und die Entwicklung von erzieherischen Präventivmaßnahmen gegen die Einnahme von verbotenen Substanzen. Seit 2018 ist die ITA vom IOC mit der Durchführung der Doping-Tests vor und während der Olympischen Spiele, der Olympischen Winterspiele, der Olympischen Jugendspiele sowie der Nachtests der eingelagerten Doping-Proben beauftragt.

Mit der Einführung der ITA als neue und zusätzlich agierende Anti-Doping-Institution und den ihr zugeschriebenen Verantwortungsbereichen musste ein Teil der Doping-Statistiken korrigiert werden. Die im Vergleich zu 2013 veränderten Zahlen bei den Olympischen Spielen in Peking 2008 und London 2012 kommen durch die vom IOC und der ITA neu durchgeführten und ausgeweiteten Nachtests zustande. Diese sind für Peking und London nun abgeschlossen; die Nachtests von den Olympischen Spielen in Rio 2016, von Tokio 2020/2021 und Paris 2024 sind noch nicht beendet (Tab. 2). Die für die Olympischen Spiele London 2012 nachgewiesenen 73 Doping-Fälle lassen sich 16 nationalen olympischen Komitees (Tab. 3) und 8 Sportdisziplinen (Tab. 4) zuweisen. Insgesamt wurden durch die aufdeckten Doping-Verstöße 31 Medaillen aberkannt und neu zugewiesen (Tab. 5).

**Tab. 2:** Doping-Statistiken bei den Olympischen Spielen 2008 bis 2024 [28–30]. Für 2008 und 2012 wurden weitere Nachtests durchgeführt (Tab. 1).

	Urin-Blutproben	Positive Tests	Nachtests	Positive Tests	Status der Nachtests
Peking 2008	4800 (IOC)	6	1053 (IOC)	54	Beendet
London 2012	5000 (IOC)	7	2727 (ITA)	73	Beendet
Rio 2016	4882 (IOC)	6	Endgültige Zahl noch nicht verfügbar (ITA)	10	Abschluss bis September 2026
Tokio 2020/21	6184 (ITA)	11	Noch keine endgültigen Zahlen verfügbar (ITA)	_	Im Prozess bis 2031
Paris 2024	6130 (ITA)	5	Noch keine endgültigen Angaben verfügbar (ITA)	_	Im Prozess bis 2034

*ITA* International Testing Agency, *IOC* Internationales Olympisches Komitee

**Tab. 3:** Positive Doping-Tests bei den Olympischen Spielen 2012 in London und Zuordnung zu nationalen olympischen Komitees [28]

Land (Nationales Olympisches Komitee)	Anzahl an positiven Tests
Russland	21
Belarus	11
Ukraine	7
Kasachstan	6
Türkei	5
Rumänien	7
Armenien	3
Aserbaidschan	3
Albanien	2
Bulgarien	2
Georgien	2
Moldawien	2
Usbekistan	2
Lettland	1
Litauen	1
Slowenien	1
*Gesamt*	*73*

**Tab. 4:** Zuordnung von positiven Doping-Tests zu Sportarten (Olympische Spiele 2012 in London; [28]).

Sportdisziplin	Anzahl an positiven Tests
Gewichtheben	36
Leichtathletik	28
Wrestling	3
Kanu	2
Boxen	1
Radfahren	1
Schwimmen	1
Volleyball	1
*Gesamt*	*73*

**Tab. 5:** Aberkennung von Medaillen bei Olympischen Spielen 2012 in London [28].

Medaille	Sportdisziplin	Anzahl
Gold	Gewichtheben	5
	Leichtathletik	2
	Ringen	1
Silber	Leichtathletik	7
	Gewichtheben	5
	Kanu	1
	Ringen	1
Bronze	Gewichtheben	8
	Leichtathletik	1
*Gesamt*		*31*

## Die Ausweitung des Anti-Doping-Netzwerks und aktuelle Herausforderungen

Seit den 1960er-Jahren wurden immer mehr Institutionen zur Bekämpfung der Doping-Problematik im Sport aufgebaut. Meist hatte sie das IOC initiiert. Zentrale Akteure waren und sind dabei das IOC Sub-Komitee Doping, die MK des IOC, die WADA, die NADAs und die ITA. Die Entstehung und Erweiterung des institutionellen Netzwerkes wurden vorangetrieben durch Doping-Missbrauch, der mitverantwortlich für Todesfälle von Athlet:innen war, durch kritische politische Positionen gegen die vom IOC intern entwickelten Anti-Doping-Strategien sowie durch Whistleblower und Berichte von Investigativjournalisten.

Die zunehmende Ausweitung des institutionellen Anti-Doping-Netzwerkes ging einher mit einer politisch initiierten Verlagerung der Anti-Doping-Strategien vom IOC an die WADA und NADAs. Dadurch sollte die Transparenz in der Doping-Bekämpfung erhöht und besser koordiniert werden. Wie sich dadurch die Statistiken über positiv getestete Athlet:innen geändert haben, ist in dem Artikel dargelegt worden und als Verweis auf die Tatsache zu verstehen, dass die Doping-Problematik bestenfalls eingegrenzt werden kann. Aber auch zur Eingrenzung des Dopings sind weiterführende politische, sportverbandspolitische, rechtliche und wissenschaftliche Unterstützungsstrategien notwendig, ebenso die Optimierung des Zusammenspiels der institutionellen Akteure im Anti-Doping-Netzwerk.

Im Mai 2026 sollen in Las Vegas (USA) die ersten *Enhanced Games *stattfinden, bei denen leistungsfördernde Medikamente und Maßnahmen ausdrücklich erlaubt sind. In diesem Kontext spielt das institutionelle Anti-Doping-Netzwerk keine Rolle. Die Gründer dieser Spiele, eine Gruppe von (Bio‑)Tech-Milliardären, haben Disziplinen aus den Sportarten Schwimmen, Leichtathletik und Gewichtheben in das Programm aufgenommen [26]. Bei den *Enhanced Games* wird auf Doping-Kontrollen verzichtet, sodass Athlet:innen ihre Leistungen im Training und Wettkampf gezielt steigern können. Diese Veranstaltung versteht sich als Gegenposition zu den Olympischen Spielen und argumentiert, dass das IOC durch seine Anti-Doping-Politik die Karriereverläufe von Athlet:innen einschränkt. Bei den *Enhanced Games *liegt es in der Verantwortung der Athlet:innen sowie in deren finanziellen Möglichkeiten, welche Doping-Präparate und -Methoden sie anwenden können. Diese Ausrichtung untergräbt nicht nur die Chancengleichheit, sondern akzeptiert auch explizit gesundheitliche Risiken. Die physische und psychische Gesundheit, die nachweislich durch die Einnahme von Doping-Mitteln gefährdet wird, wird bei den *Enhanced Games *der Eigenverantwortung der Athlet:innen zugeschrieben. Letztlich wird auch dazu aufgefordert, bewusst gegen das Arzneimittelgesetz zu verstoßen, wodurch für Athlet:innen rechtliche Konsequenzen entstehen können. Zur Kompensation der persönlichen und rechtlichen Gefahrenlage werden Preisgelder in Höhe von 500.000 US-Dollar ausgelobt. Für einen neuen Weltrekord wird eine Belohnung von einer Million US-Dollar ausgelobt. Bei den Olympischen Spielen werden grundsätzlich keine Preisgelder und Prämien für Rekorde gezahlt.

In gemeinsamen Stellungnahmen haben IOC, WADA, ITA und NADAs die *Enhanced Games *als indiskutabel und als Gegenmodell zu einem ethisch und gesundheitlich verantwortbaren Sportprinzip bezeichnet. Es darf aber nicht nur bei dieser Verurteilung bleiben. Gezielte Initiativen müssen unternommen werden, um den *Enhanced Games *ihre Bedeutung zu nehmen. Ein Ansatzpunkt dafür wären Informations- und Erziehungskampagnen über das grundlegend falsche Verständnis der Gründer der *Enhanced Games *zum sportlichen Wettkampf. Als Zielgruppe für diese Initiative sollten zunächst Athlet:innen angesprochen werden. Sie sind nicht nur die aktiven Hauptakteur:innen, sondern auch diejenigen, die die größten gesundheitlichen und rechtlichen Risiken eingehen. Die Einführung dieser Maßnahmen wäre ein neues und bedeutsames Aufgabengebiet der Athletenvertretungen im IOC, in der WADA, in den NADAs und in der ITA. Es würde die Rolle der Athlet:innen als Vorbildfunktion für eine verantwortbare Gesundheitspolitik stärken, die über den olympischen Sport auch auf den Freizeit- und Breitensport ausstrahlen kann. Diese gesundheitspolitische Ausrichtung ist ein wichtiges Element für die Bewerbungen der Länder um die Ausrichtung der Olympischen Spiele.

## Data Availability

Alle dieser Arbeit zugrunde liegenden Daten sind in diesem Artikel enthalten.

## Footnotes

1 Krüger et al. (siehe [16]) weisen in ihrer Publikation *Doping und Anti-Doping in der Bundesrepublik Deutschland 1950 bis 2007 *aber darauf hin, dass „die Sportmedizin dank ihrer medizinischen und präventiven Ausrichtung [auch] gleichzeitig eine tragende Rolle beim Anti-Doping“ spielte.

2 Das Gesamtbudget der WADA belief sich 2025 auf 57,5 Mio. US-Dollar. Von den 50 % der Kostenübernahme durch die Regierungen entfielen 47,5 % auf Europa. Amerika (Nord‑, Mittel- und Südamerika) waren mit 29 % beteiligt, Asien mit 20,46 % sowie Ozeanien und Afrika mit jeweils 2,54 %.

3 Es handelt sich um Julija Stepanowa, ehemalige russische 800-Meter-Läuferin, die 2013 selbst des Dopings überführt worden war (2-jährige Sperre), ihren Ehemann Witali Stepanow, damaliger Mitarbeiter der RUSADA, und um Girgori Rodtschenkow, der sogar Leiter der RUSADA war. Während Stepanowa unter großem persönlichen Risiko verdeckte Filmaufnahmen mit Treffen von Spitzenathlet:innen und Funktionären aufgezeichnet hatte, informierten Stepanow und Rodtschenkow die internationale Presse über die Doping-Strategien Russlands.
